# Supplementary material for: Association of pharmacotherapy with all-cause mortality among patients with irritable bowel syndrome
Source: Commun Med (Lond). 2026 Apr 8;6:176. doi: 10.1038/s43856-026-01498-6 (PMC13061985; doi:10.1038/s43856-026-01498-6)
Supplement: Supplementary file 2 — Supplementary material [file 43856_2026_1498_MOESM2_ESM.pdf]

Supplementary Materials

TABLE OF CONTENT

Table of Contents

Title Page....2
Supplementary Table 1....3
Supplementary Table 1a. Eligibility criteria and exposure definitions....3
Supplementary Table 1b. Secondary outcomes definitions....3
Supplementary Table 1c. Definition of negative control outcomes....4
Supplementary Table 2. Mean follow-up time of all medication categories. Cumulative mortality was assessed using Kaplan–Meier survival analysis, and Cox proportional hazards models were employed to compare time-to-event mortality rates, accounting for censored data and variable follow-up durations from the index event....4
Supplementary Figure 1: Survival probability curves....5
Supplementary Figure 2: Secondary Outcomes....6
Supplementary Figure 3: Mortality rate across various refill counts....6
Supplementary Figure 4: Negative control outcomes....7

Title Page

Association of pharmacotherapy with all-cause mortality among patients with irritable bowel syndrome

Supplementary Table 1

Supplementary Table 1a. Eligibility criteria and exposure definitions.

Variable	Values	Name	Codes
Diagnose of IBS	Binary: present/absent	Irritable bowel syndrome<br>Irritable bowel syndrome with constipation<br>Mixed irritable bowel syndrome<br>Irritable bowel syndrome without diarrhea<br>Irritable bowel syndrome with diarrhea<br>Other irritable bowel syndrome	ICD10 code: K58<br>ICD10 code: K58.1<br>ICD10 code: K58.2<br>ICD10 code: K58.9<br>ICD10 code: K58.0<br>ICD10 code: K58.8
Initiate use of TCAs at baseline	Binary: present/absent	Nortriptyline<br>Amitriptyline<br>Trimipramine<br>Desipramine<br>Doxepin<br>Imipramine	RXNORM:7531<br>RXNORM:704<br>RXNORM:10834<br>RXNORM:3247<br>RXNORM:3638<br>RXNORM:5691
Initiate use of SSRIs at baseline	Binary: present/absent	Escitalopram<br>Citalopram<br>Fluoxetine<br>Fluvoxamine<br>Sertraline<br>Paroxetine	RXNORM:321988<br>RXNORM:2556<br>RXNORM:4493<br>RXNORM:42355<br>RXNORM:36437<br>RXNORM:32937
Initiate use of SNRIs at baseline	Binary: present/absent	Duloxetine<br>Venlafaxine<br>Milnacipran	RXNORM:72625<br>RXNORM:39786<br>RXNORM:588250
Initiate use of mirtazapine at baseline	Binary: present/absent	Mirtazapine	RXNORM:15996
No prescription for any antidepressant medications	Binary: present/absent	Any antidepressants	ATC: N06A

Supplementary Table 1b. Secondary outcomes definitions.

Variable	ICD10 code	Values
serotonin syndrome	G90.81	Binary: present/absent
other specified extrapyramidal and movement disorders	G25.89	Binary: present/absent
suicidal ideation	R45.851	Binary: present/absent
pneumonitis due to solids and liquids	J69	Binary: present/absent

overweight and obesity	E66	Binary: present/absent
melena	K92.1	Binary: present/absent
gastrointestinal hemorrhage unspecified	K92.2	Binary: present/absent
hematemesis	K92.0	Binary: present/absent
acute gastric ulcer with hemorrhage	K25.0	Binary: present/absent
acute duodenal ulcer with hemorrhage	K26	Binary: present/absent
acute peptic ulcer with hemorrhage	K27.0	Binary: present/absent
Cardiovascular conditions such as hypertensive diseases	I10-I15	Binary: present/absent
heart failure	I50	Binary: present/absent
ischemic heart disease	I20-I25	Binary: present/absent
other cardiac arrhythmias	I49	Binary: present/absent
cerebral infarction	I63	Binary: present/absent
unspecified falls<br>initial encounter<br>subsequent encounter<br>Unspecified fall, sequela<br>repeated falls	W19<br>W19.XXXA<br>W19.XXXD<br>W19.XXXS<br>R29.6	Binary: present/absent

ICD-10: International Classification of Diseases, Tenth Revision (ICD-10)

Supplementary Table 1c. Definition of negative control outcomes

Variable	Values	ICD10 code
blepharitis	Binary: present/absent	H01.0
frostbite	Binary: present/absent	T33-T34
acute appendicitis	Binary: present/absent	K35.2
nummular dermatitis	Binary: present/absent	L30.0
eczematous dermatitis of the eyelid	Binary: present/absent	H01.13
retinal detachment	Binary: present/absent	H33
protozoal diseases	Binary: present/absent	B50-B65

ICD-10: International Classification of Diseases, Tenth Revision (ICD-10)

Supplementary Table 2. Mean follow-up time of all medication categories.

Cumulative mortality was assessed using Kaplan–Meier survival analysis, and Cox proportional hazards models were employed to compare time-to-event mortality rates, accounting for censored data and variable follow-up durations from the index event.

Medication categories	Users follow up time (years SD)	Non-users follow up time (years SD)
Antidepressants	5.1 ± 3.5	4.3 ± 3.6
SSRIs	5.0 ± 3.5	4.3 ± 3.5
TCAs	5.2 ± 3.5	4.2 ± 3.4
SNRIs	4.8 ± 3.3	4.2 ± 3.3
Mirtazapine	4.4 ± 3.1	3.9 ± 3.1
Antispasmodics	4.6 ± 3.6	4.8 ± 3.6

TCA: tricyclic antidepressant; SSRI: selective serotonin reuptake inhibitor; SNRI: serotonin-norepinephrine reuptake inhibitor

Supplementary Figure 1: Survival probability curves

For patients with IBS-D comparing antispasmodic users (dicyclomine and hyoscyamine) with mu receptor agonist users (loperamide and diphenoxylate). IBS-D: Irritable bowel syndrome with diarrhea

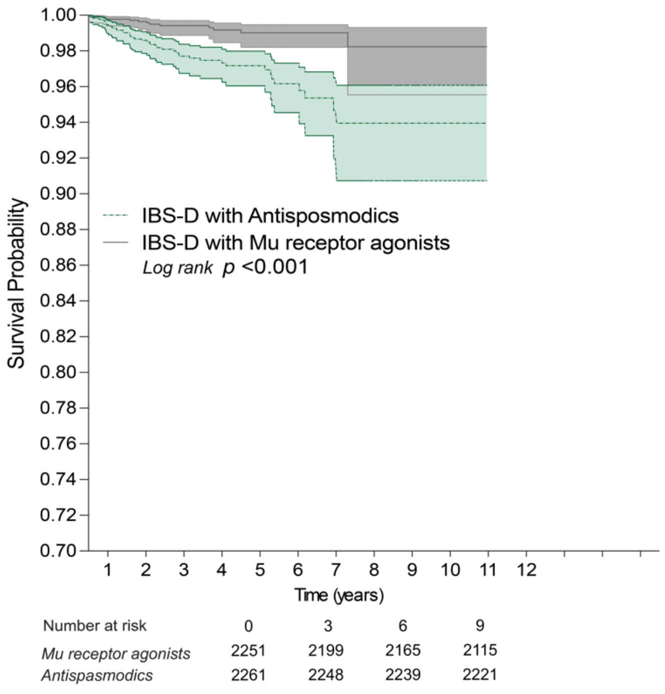

Supplementary Figure 2: Secondary Outcomes

Comparison of secondary outcomes and hazard ratio between patients with IBS using antidepressants and non-users. Propensity score matching was applied to balance baseline characteristics between antidepressant users and nonusers. HRs were estimated using Kaplan–Meier analysis, incorporating daily time intervals and censoring to evaluate the probability of outcomes. HR: Hazard ratio; CI: Confidence interval

Secondary outcomes	HR (95% CI)	P-value
Inpatient encounter	1.21 (1.16, 1.27)	< 0.001
Hypertensive diseases	1.43 (1.36, 1.51)	< 0.001
Cardiac arrhythmias	1.46 (1.34, 1.58)	< 0.001
Heart failure and ischemic heart disease	1.36 (1.23, 1.50)	< 0.001
Aspiration pneumonitis	2.43 (1.55, 3.81)	< 0.001
Fall	1.71 (1.54, 1.89)	< 0.001
Stroke	1.75 (1.37, 2.24)	< 0.001
Serotonin syndrome	1.53 (1.04, 2.23)	0.0274
Suicidal ideation	5.19 (4.21, 6.40)	< 0.001
Gastrointestinal bleeding	1.47 (1.34, 1.61)	< 0.001
Obesity	1.84 (1.75, 1.93)	< 0.001

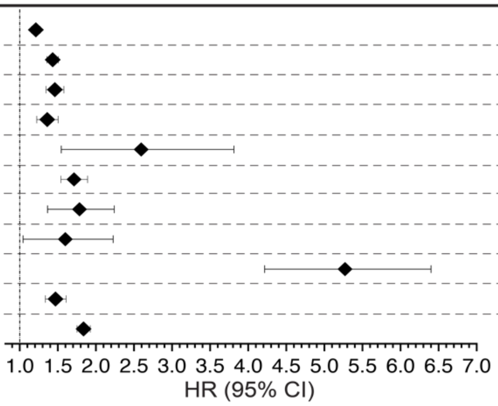

Supplementary Figure 3: Mortality rate across various refill counts

Comparison of mortality in the study population with IBS between propensity score-matched antidepressant users and non-users across various refill counts (2, 4, 8, 12, 16, and 20 refills). HRs were calculated using Kaplan–Meier analysis to estimate the probability of the outcome at daily time intervals, with censoring applied. HR: Hazard ratio; CI: Confidence interval

Number of refills	HR (95% CI)	P-value
20-refills	1.91 (1.68, 2.17)	< 0.001
16-refills	1.91 (1.70, 2.16)	< 0.001
12-refills	1.70 (1.53, 1.90)	< 0.001
8-refills	1.66 (1.50, 1.82)	< 0.001
4-refills	1.53 (1.42, 1.67)	< 0.001
2-refills	1.39 (1.27, 1.51)	< 0.001

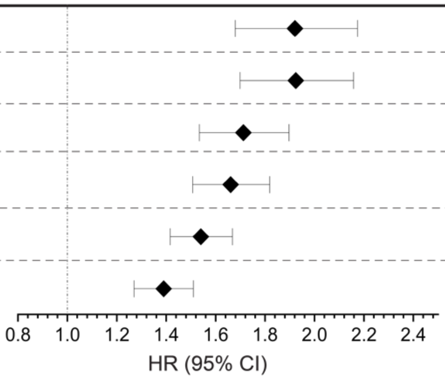

Supplementary Figure 4: Negative control outcomes

HR: Hazard ratio; CI: Confidence interval

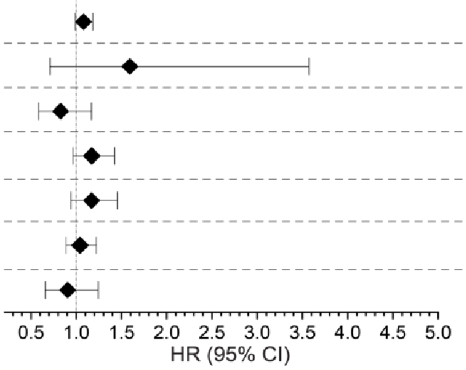

Negative control outcomes	HR (95% CI)	P-value
Blepharitis	1.08 (0.99, 1.18)	0.108
Frostbite	1.59 (0.71, 3.57)	0.254
Acute appendicitis	0.83 (0.58, 1.17)	0.285
Nummular dermatitis	1.17 (0.96, 1.42)	0.108
Eczematous dermatitis of eyelid	1.17 (0.94, 1.46)	0.154
Retinal detachments	1.04 (0.89, 1.22)	0.610
Protozoal diseases	0.90 (0.66, 1.24)	0.541
