# Supplementary material for: Association of pharmacotherapy with all-cause mortality among patients with irritable bowel syndrome
Source: Commun Med (Lond). 2026 Apr 8;6:176. doi: 10.1038/s43856-026-01498-6 (PMC13061985; doi:10.1038/s43856-026-01498-6)
Supplement: Supplementary file 3 — Description of Additional Supplementary files [file 43856_2026_1498_MOESM3_ESM.docx]

**Description of Additional Supplementary Files**

Supplementary Data1. Inclusion and exclusion criteria.

Supplementary Data 2: Definitions of covariates.

Supplementary Data 3. Baseline Demographic and Clinical Characteristics of antidepressant users and non-users

Supplementary Data 4. Baseline Demographic and Clinical Characteristics of SSRI users and non-users

Supplementary Data 5. Baseline Demographic and Clinical Characteristics of TCA users and non-users

Supplementary Data 6. Baseline Demographic and Clinical Characteristics of SNRI users and non-users

Supplementary Data 7. Baseline Demographic and Clinical Characteristics of mirtazapine users and non-users

Supplementary Data 8. Baseline Demographic and Clinical Characteristics of antispasmodic users and non-users

Supplementary Data 9: Active comparison of all-cause mortality risk and hazard ratios among patients with IBS, evaluating antispasmodic users against users of antidepressants, including SSRIs, TCAs, SNRIs, and mirtazapine.
