# Supplementary material for: Association of pharmacotherapy with all-cause mortality among patients with irritable bowel syndrome
Source: Commun Med (Lond). 2026 Apr 8;6:176. doi: 10.1038/s43856-026-01498-6 (PMC13061985; doi:10.1038/s43856-026-01498-6)
Supplement: Supplementary file 4 — Supplementary Data 1-9 [file 43856_2026_1498_MOESM4_ESM.pdf]

Supplementary Data1. Inclusion and exclusion criteria. 1
Supplementary Data 2: Definitions of covariates. 4
Supplementary Data 3. Baseline Demographic and Clinical Characteristics of antidepressant users and non-users 6
Supplementary Data 4. Baseline Demographic and Clinical Characteristics of SSRI users and non-users 9
Supplementary Data 5. Baseline Demographic and Clinical Characteristics of TCA users and non-users 12
Supplementary Data 6. Baseline Demographic and Clinical Characteristics of SNRI users and non-users 15
Supplementary Data 7. Baseline Demographic and Clinical Characteristics of mirtazapine users and non-users 18
Supplementary Data 8. Baseline Demographic and Clinical Characteristics of antispasmodic users and non-users 21
Supplementary Data 9: Active comparison of all-cause mortality risk and hazard ratios among patients with IBS, evaluating antispasmodic users against users of antidepressants, including SSRIs, TCAs, SNRIs, and mirtazapine. 24

Supplementary Data1. Inclusion and exclusion criteria.

Comparing the antidepressants users with the antidepressant nonusers and Target trial emulation for risk of mortality outcomes in patients with comorbid IBS using EHR data and analytics functions from the TriNetX Analytics Platform.

Protocol	Inclusion criteria	Exclusion criteria
Eligibility criteria for antidepressant users	▪ participants aged 18 to 65<br>▪ With diagnosed of IBS from January 1, 2005, to January 1, 2023,<br>▪ were prescribed these antidepressants including selective serotonin reuptake inhibitors* (SSRIs), tricyclic antidepressants* (TCAs), selective serotonin and norepinephrine reuptake inhibitors* (SNRIs), and mirtazapine<br>▪ should receive antidepressants after IBS diagnosis<br>▪ with at least two refills of each medication during this period.<br><br>*SSRIs: citalopram, sertraline, escitalopram, fluoxetine, fluvoxamine, paroxetine<br>*TCAs: amitriptyline, imipramine, desipramine, nortriptyline, doxepin, trimipramine<br>*SNRIs: venlafaxine, duloxetine, milnacipran	No encounter with death before the index event.<br><br>No antidepressant use history before the index event.
Treatment strategies for patients with IBS	▪ For antidepressant users:<br>o Users: Initiate use of antidepressants at index event<br>o Non-users: Never take any antidepressant medications<br><br>▪ For SSRI users:<br>o Users: Initiate use of SSRIS at index event<br>o Non-users: Never take any antidepressant medications<br><br>▪ For SNRI users:<br>o Users: Initiate use of SNRIs at index event<br>o Non-users: Never take any antidepressant medications<br><br>▪ For TCA users:<br>o Users: Initiate use of TCAs at index event<br>o Non-users: Never take any antidepressant medications<br><br>▪ For mirtazapine users:<br>o Users: Initiate use of mirtazapine at index event<br>o Non-users: Never take any antidepressant medications	
Treatment strategies for target trial emulation for patients with IBS	▪ For the target trial comparing antispasmodic user's vs antidepressant users<br>o Initiate use of antispasmodic (hyoscyamine and dicyclomine) at index event and never take any antidepressant medications<br>o Initiate use of antidepressants at index event<br><br>▪ For the target trial comparing SSRI user's vs antidepressant users	

	o Initiate antispasmodic use at the index event and never take antidepressant medications<br>o Initiate use of SSRIs at index event<br>▪ For the target trial comparing TCA user's vs antidepressant users<br>o Initiate antispasmodic use at the index event and never take antidepressant medications<br>o Initiate use of TCAs at index event<br><br>▪ For the target trial comparing SNRI user's vs antidepressant users<br>o Initiate use of antispasmodic at index event and not taking any antidepressant medications<br>o Initiate use of SNRIs at index event<br><br>▪ For the target trial comparing mirtazapine user's vs antidepressant users<br>o Initiate use of antispasmodic at index event and not taking any antidepressant medications<br>o Initiate use of mirtazapine at index event	
Treatment strategies for patients with IBS-D	▪ For antidepressant users:<br>o Users: Initiate use of antidepressants at index event<br>o Non-users: Never take any antidepressant medications<br><br>▪ For antispasmodics users:<br>o Users: Initiate use of antispasmodics at index event<br>o Non-users: Never take any antidepressant and antispasmodics medications<br><br>▪ For eluxadoline users:<br>o Users: Initiate use of eluxadoline at index event<br>o Non-users: Never take any antidepressant and eluxadoline medications<br><br>▪ For bile acid sequestrants users:<br>o Users: Initiate use of bile acid sequestrants (cholestyramine/colestipol) at index event<br>o Non-users: Never take any antidepressant and bile acid sequestrants medications<br><br>▪ For rifaximin users:<br>o Users: Initiate use of rifaximin at index event<br>o Non-users: Never take any antidepressant and rifaximin medications<br><br>▪ For loperamide users:<br>o Users: Initiate use of loperamide at index event<br>o Non-users: Never take any antidepressant and loperamide medications<br><br>▪ For diphenoxylate users:<br>o Users: Initiate use of rifaximin at index event	

	○ Non-users: Never take any antidepressant and diphenoxylate medications	
Treatment strategies for patients with IBS-C	▪ For antidepressant users:<br>○ Users: Initiate use of antidepressants at index event<br>○ Non-users: Never take any antidepressant medications<br><br>▪ For antispasmodics users:<br>○ Users: Initiate use of antispasmodics at index event<br>○ Non-users: Never take any antidepressant and antispasmodics medications<br><br>▪ For PEG-3350 users:<br>○ Users: Initiate use of PEG-3350 at index event<br>○ Non-users: Never take any antidepressant and PEG-3350 medications<br><br>▪ For secretagogues* users:<br>○ Users: Initiate use of secretagogues at index event<br>○ Non-users: Never take any antidepressant and secretagogues medications<br>*tenapanor, plecanatide, lubiprostone, and linaclotide	
Treatment assignment	▪ Individuals are randomly assigned to a treatment strategy at baseline. Individuals will be aware of the assigned treatment strategies.<br>▪ Individuals are assigned to the strategy compatible with their prescription and assumed randomization by propensity-score matching for baseline covariates.	
Casual contrast of interest	Intention-to-treat	
Follow-up	Follow-up for each individual will start 6 months after the treatment assignment and end on day of outcome, death, loss to follow-up, or 15 years after baseline, whichever occurs first.	

Supplementary Data 2: Definitions of covariates.

Variable	Value		Coding terminology
Age at Index	Continuous	AI	Demographics

White	Binary: present/absent	2106-3	Demographics
Female	Binary: present/absent	F	Demographics
Black or African American	Binary: present/absent	2054-5	Demographics
Hispanic or Latino	Binary: present/absent	2135-2	Demographics
Asian	Binary: present/absent	2028-9	Demographics
Neoplasms	Binary: present/absent	C00-D49	ICD-10
Noninfective enteritis and colitis	Binary: present/absent	K50-K52	ICD-10
Nicotine dependence	Binary: present/absent	F17	ICD-10
Type 2 diabetes mellitus	Binary: present/absent	E11	ICD-10
Generalized anxiety disorder	Binary: present/absent	F41.1	ICD-10
Personal history of nicotine dependence	Binary: present/absent	Z87.891	ICD-10
Bipolar disorder	Binary: present/absent	F31	ICD-10
Other specified diseases of liver	Binary: present/absent	K76.89	ICD-10
Persons with potential health hazards related to socioeconomic and psychosocial circumstances	Binary: present/absent	Z55-Z65	ICD-10
Chronic ischemic heart disease	Binary: present/absent	I25	ICD-10
Alcohol related disorders	Binary: present/absent	F10	ICD-10
Chronic kidney disease (CKD)	Binary: present/absent	N18	ICD-10
Cannabis related disorders	Binary: present/absent	F12	ICD-10
Somatoform disorders	Binary: present/absent	F45	ICD-10
Major depressive disorder, recurrent, moderate	Binary: present/absent	F33.1	ICD-10
Heart failure	Binary: present/absent	I50	ICD-10
Type 1 diabetes mellitus	Binary: present/absent	E10	ICD-10
Opioid related disorders	Binary: present/absent	F11	ICD-10
Atrial fibrillation and flutter	Binary: present/absent	I48	ICD-10
Eating disorders	Binary: present/absent	F50	ICD-10
Phobic anxiety disorders	Binary: present/absent	F40	ICD-10

Major depressive disorder, recurrent, unspecified	Binary: present/absent	F33.9	ICD-10
Obsessive-compulsive disorder	Binary: present/absent	F42	ICD-10
Fibrosis and cirrhosis of liver	Binary: present/absent	K74	ICD-10
Specific personality disorders	Binary: present/absent	F60	ICD-10
Major depressive disorder, recurrent severe without psychotic features	Binary: present/absent	F33.2	ICD-10
Major depressive disorder, recurrent, mild	Binary: present/absent	F33.0	ICD-10
Chronic hepatitis, not elsewhere classified	Binary: present/absent	K73	ICD-10
Hepatic failure, not elsewhere classified	Binary: present/absent	K72	ICD-10
Major depressive disorder, recurrent, severe with psychotic symptoms	Binary: present/absent	F33.3	ICD-10
Other specified inflammatory liver diseases	Binary: present/absent	K75.89	ICD-10
BMI (Body mass index)	Binary: present/absent	9083	TNX Curated
Cholesterol in LDL [Mass/volume] in Serum or Plasma	Binary: present/absent	9002	TNX Curated
Hemoglobin A1c/Hemoglobin.total in Blood	Binary: present/absent	9037	TNX Curated
Emergency Department Services	Binary: present/absent	1013711	CPT
Preventive Medicine Services	Binary: present/absent	1013829	CPT
OPIOID ANALGESICS	Binary: present/absent	CN101	VA
Benzodiazepine derivative sedatives/hypnotics	Binary: present/absent	CN302	VA
Polyethylene glycol 3350	Binary: present/absent	221147	VA
Antipsychotics	Binary: present/absent	CN700	RxNorm
linaclotide	Binary: present/absent	1307404	RxNorm
loperamide	Binary: present/absent	6468	RxNorm
diphenoxylate	Binary: present/absent	3500	RxNorm
lubiprostone	Binary: present/absent	623033	RxNorm
bismuth subsalicylate	Binary: present/absent	19478	RxNorm
Antimuscarinic/antipasmodic combinations	Binary: present/absent	GA802	VA
plecanatide	Binary: present/absent	1873752	RxNorm
eluxadoline	Binary: present/absent	1653781	RxNorm

prucalopride	Binary: present/absent	2107310	RxNorm
tegaserod	Binary: present/absent	139778	RxNorm
tenapanor	Binary: present/absent	2199674	RxNorm

ICD-10: International Classification of Diseases, Tenth Revision (ICD-10); RxNORM: Medical Prescription Normalized Medical Prescription; ATC: Anatomical Therapeutic Chemical; CPT: Current Procedural Terminology; VA: Veterans Affairs Drug Classifications

Supplementary Data 3. Baseline Demographic and Clinical Characteristics of antidepressant users and non-users

	Before PSM			After PSM		
	Antidepressant users N=196036	Non-users N=331046	SMD	Antidepressant users N=120341	Non-users N=120341	SMD
Demographics (N)						
Age at Index (mean, SD)	39.27, 12.5	37.03, 13.2	0.175	38.13, 12.6	38.26, 12.9	0.010
Female	152316	218892	0.260	89541	88998	0.010
White	148629	205108	0.303	88238	89031	0.015
Black or African American	13639	22008	0.012	7889	7944	0.002
Hispanic or Latino	10796	19554	0.017	6803	7269	0.017
Asian	2560	11347	0.140	2089	2166	0.005
Comorbidities (N)						
Neoplasms	55152	43311	0.379	25272	25989	0.015
Other specified diseases of liver	8477	4043	0.190	2906	2972	0.004
Fibrosis and cirrhosis of liver	2351	1146	0.098	785	772	0.001
Hepatic failure, not elsewhere classified	1036	330	0.077	268	249	0.003
Chronic hepatitis, not elsewhere classified	529	668	0.014	254	263	0.002
Other specified inflammatory liver diseases	103	36	0.023	29	23	0.003
Type 2 diabetes mellitus	21273	10462	0.305	7754	7728	0.001
Type 1 diabetes mellitus	3734	1572	0.132	1189	1115	0.006
Chronic ischemic heart disease	8070	3507	0.193	2591	2463	0.007
Heart failure	4596	1536	0.160	1284	1208	0.006
Atrial fibrillation and flutter	3071	1552	0.110	1150	1052	0.009
Chronic kidney disease (CKD)	5993	2699	0.163	1926	1864	0.004
Nicotine dependence	32021	13795	0.409	11100	10942	0.005

Opioid related disorders	5883	1046	0.211	1125	953	0.015
Alcohol related disorders	8511	2586	0.227	2303	2094	0.013
Cannabis related disorders	6829	2189	0.199	1875	1716	0.011
Personal history of nicotine dependence	18101	7651	0.300	6196	6029	0.006
Generalized anxiety disorder	35007	7064	0.543	7909	6821	0.038
Phobic anxiety disorders	5392	1051	0.199	1128	963	0.015
Eating disorders	4310	1036	0.170	995	922	0.007
Specific personality disorders	4798	673	0.197	746	586	0.018
Bipolar disorder	13649	3868	0.297	3387	3105	0.014
Persons with potential health hazards related to socioeconomic and psychosocial circumstances	10175	3198	0.246	2745	2580	0.009
Somatoform disorders	5208	1562	0.177	1401	1265	0.011
Obsessive-compulsive disorder	4696	750	0.192	924	730	0.020
Major depressive disorder, recurrent, moderate	11286	1230	0.316	1614	1186	0.033
Major depressive disorder, recurrent, unspecified	6895	822	0.242	1062	782	0.027
Major depressive disorder, recurrent severe without psychotic features	6338	612	0.237	827	584	0.026
Major depressive disorder, recurrent, mild	3886	405	0.183	550	385	0.022
Major depressive disorder, recurrent, severe with psychotic symptoms	1183	110	0.101	149	102	0.012
Laboratory values (mean, SD)						
BMI (body mass index)	29.56, 8.2	27.52, 7.2	0.265	28.88, 7.8	27.98, 7.4	0.118
Cholesterol (mg/dL)	105.67, 37.4	105.91, 34.7	0.007	106.42, 36.4	105.44, 34.6	0.028
Hemoglobin A1C	5.95, 1.8	5.98, 1.9	0.016	5.97, 1.9	5.92, 1.8	0.026
Visits (N)						

Preventive Medicine Services	64878	45864	0.466	30885	32340	0.027
Emergency Department Services	68973	45305	0.517	29406	30301	0.017
Medications (N)						
Opioid analgesics	114035	69824	0.819	51847	54519	0.045
Benzodiazepine derivative	98318	43797	0.865	38282	38944	0.012
polyethylene glycol 3350	46770	22080	0.492	16826	16961	0.003
Antipsychotics	28270	4779	0.495	5488	4602	0.037
linaclotide	12200	4063	0.266	3621	3518	0.005
loperamide	10100	3654	0.234	2995	2873	0.007
diphenoxylate	6360	1931	0.195	1850	1690	0.011
lubiprostone	5966	1670	0.193	1632	1472	0.012
Antimuscarinic/antispasmodic combinations	2089	593, 0	0.113	620	574	0.005
bismuth subsalicylate	1887	735, 0	0.097	562	566	<0.001
plecanatide	1345	505, 0	0.083	403	387	0.002
eluxadoline	1255	349, 0	0.088	347	336	0.002
tegaserod	472	189, 0	0.048	179	164	0.003
prucalopride	666	201, 0	0.062	162	160	<0.001
tenapanor	21	10, 0	0.009	10	10	<0.001

SMD: standardized mean difference; PSM: propensity score matching

Supplementary Data 4. Baseline Demographic and Clinical Characteristics of SSRI users and non-users

	Before PSM			After PSM		
	SSRI users N=130311	Non-users N=332756	SMD	SSRI users N=88584	Non-users N=88584	SMD
Demographics (N)						
Age at Index (mean, SD)	38.41, 12.5	37.06, 13.2	0.105	37.72	37.99, 12.8	0.021
Female	101543	220238	0.264	66829	66201	0.016
White	100838	206593	0.338	66801	67749	0.025
Black or African American	8342	22715	0.017	5519	5616	0.005
Hispanic or Latino	6740	19257	0.027	4681	4811	0.007
Asian	1445	1118	0.153	1219	1221	<.002
Comorbidities (N)						
Neoplasms	36484	43179	0.379	19764	20424, 23	0.018
Other specified diseases of liver	5443	3983	0.185	2359	2293	0.005
Fibrosis and cirrhosis of liver	1445	1145	0.090	642	616	0.004
Hepatic failure, not elsewhere classified	719	330	0.080	249	213	0.008
Chronic hepatitis, not elsewhere classified	285	668	0.004	154	145	0.002
Other specified inflammatory liver diseases	72	36	0.024	29	22	0.005
Type 2 diabetes mellitus	13127	10531	0.281	6010	5975	0.002
Type 1 diabetes mellitus	2410	1564	0.129	964	914	0.006
Chronic ischemic heart disease	4979	3512	0.180	2067	2008	0.004
Heart failure	2923	1550	0.154	1076	1015	0.006
Atrial fibrillation and flutter	1990	1566	0.106	892	848	0.005
Chronic kidney disease (CKD)	3704	2701	0.152	1558	1498	0.005
Nicotine dependence	20989	13813	0.404	9047	9034	0.000
Opioid related disorders	3647	1044	0.202	940	815	0.014
Alcohol related disorders	5919	2577	0.236	1974	1823	0.012
Cannabis related disorders	4556	2186	0.200	1537	1456	0.007
Personal history of nicotine dependence	11760	7623	0.295	4967	4912	0.003

Generalized anxiety disorder	26600	7057	0.604	7387	6410	0.041
Phobic anxiety disorders	3986	1057	0.214	1054	887	0.018
Eating disorders	3131	1036	0.182	923	817	0.012
Specific personality disorders	3396	681	0.205	722	528	0.026
Bipolar disorder	8869	3893	0.291	2946	2598	0.023
Persons with potential health hazards related to socioeconomic and psychosocial circumstances	7320	3160	0.264	2372	2196	0.013
Somatoform disorders	3439	1559	0.176	1129	1065	0.007
Obsessive-compulsive disorder	3809	749	0.218	902	690	0.025
Major depressive disorder, recurrent, moderate	8470	1231	0.341	1460	1109	0.033
Major depressive disorder, recurrent, unspecified	5016	821	0.256	1005	728	0.032
Major depressive disorder, recurrent severe without psychotic features	4626	614	0.251	778	535	0.032
Major depressive disorder, recurrent, mild	2980	412	0.199	528	368	0.025
Major depressive disorder, recurrent, severe with psychotic symptoms	878	110	0.108	137	91	0.014
Laboratory values (mean, SD)						
BMI (body mass index)	29.52, 8.14	27.57, 7.2	0.253	29.03, 7.8	28.04, 7.5	0.129
Cholesterol (mg/dL)	105.41, 36.9	105.96, 34.7	0.015	106.19, 36.5	105.11, 34.7	0.030
Hemoglobin A1C	5.90, 1.8	5.98, 1.9	0.042	5.91, 1.8	5.88, 1.7	0.023
Visits (N)						
Preventive Medicine Services	47888	45843	0.548	26367	27471	0.027
Emergency Department Services	46113	45484	0.522	23312	23942	0.016
Medications (N)						
Opioid analgesics	74590	70614	0.794	40854	43161	0.052
Benzodiazepine derivative sedatives/hypnotics	65391	43875	0.867	31667	32546	0.021
polyethylene glycol 3350	30225	22057	0.478	13455	13708	0.008
Antipsychotics	19219	5045	0.499	5221	4591	0.031
linaclotide	7651	4099	0.253	2953	2919	0.002
loperamide	6779	3697	0.236	2465	2394	0.005
diphenoxylate	4276	1966	0.196	1544	1489	0.005

lubiprostone	3823	1683	0.188	1330	1262	0.006
Antimuscarinic/antispasmodic combinations	1392	600	0.113	549	523	0.004
bismuth subsalicylate	1246	730	0.096	470	457	0.002
plecanatide	865	499	0.081	326	330	0.001
eluxadoline	826	348	0.087	297	286	0.002
tegaserod	311	190	0.047	153	139	0.004
prucalopride	355	196	0.053	119	116	0.001
tenapanor	10	10	0.006	10	10	0.000

SMD: standardized mean difference; PSM: propensity score matching

Supplementary Data 5. Baseline Demographic and Clinical Characteristics of TCA users and non-users

	Before PSM			After PSM		
	TCA users N=130311	Non-users N=332756	SMD	TCA users N=88584	Non-users N=88584	SMD
Demographics (N)						
Age at Index (mean, SD)	39.56	37.09	0.191	38.77	38.7	0.003
Female	36339	217107	0.262	28559	28049	0.031
White	34305	203355	0.248	27391	28135	0.045
Black or African American	4426	22554	0.095	3337	3184	0.014
Hispanic or Latino	2917	18921	0.020	2334	2355	0.002
Asian	735	11109	0.117	694	635	0.012
Comorbidities (N)						
Neoplasms	14384	42386	0.443	9812	9863	0.003
Other specified diseases of liver	2748	3896	0.256	1507	1432	0.010
Fibrosis and cirrhosis of liver	731	1135	0.126	384	340	0.012
Hepatic failure, not elsewhere classified	291	326	0.087	135	128	0.003
Chronic hepatitis, not elsewhere classified	178	667	0.033	113	97	0.008
Other specified inflammatory liver diseases	28	36	0.026	13	11	0.003
Type 2 diabetes mellitus	5915	10373	0.358	3387	3312	0.007
Type 1 diabetes mellitus	1177	1539	0.170	548	533	0.003
Chronic ischemic heart disease	2284	3428	0.228	1220	1125	0.015
Heart failure	1355	1530	0.190	655	632	0.005
Atrial fibrillation and flutter	854	1538	0.128	461	468	0.002
Chronic kidney disease (CKD)	1889	2670	0.211	969	896	0.012
Nicotine dependence	8308	13539	0.448	4858	4785	0.006
Opioid related disorders	1952	1024	0.263	634	583	0.011
Alcohol related disorders	1935	2513	0.220	920	852	0.012
Cannabis related disorders	1895	2121	0.227	884	837	0.008
Personal history of nicotine dependence	4699	7365	0.330	2714	2644	0.007

Generalized anxiety disorder	7792	7028	0.514	3518	3344	0.016
Phobic anxiety disorders	1390	1044	0.210	540	494	0.010
Eating disorders	908	1031	0.155	376	376	< 0.001
Specific personality disorders	1252	673	0.209	362	332	0.008
Bipolar disorder	3499	3856	0.314	1504	1407	0.013
Persons with potential health hazards related to socioeconomic and psychosocial circumstances	2582	3090	0.261	1192	1119	0.011
Somatoform disorders	1902	1542	0.244	782	756	0.005
Obsessive-compulsive disorder	878	746	0.162	341	314	0.008
Major depressive disorder, recurrent, moderate	2926	1228	0.333	782	699	0.016
Major depressive disorder, recurrent, unspecified	1879	817	0.263	526	463	0.015
Major depressive disorder, recurrent severe without psychotic features	1747	612	0.258	398	359	0.010
Major depressive disorder, recurrent, mild	940	412	0.184	242	228	0.005
Major depressive disorder, recurrent, severe with psychotic symptoms	324	108	0.110	68	55	0.009
Laboratory values (mean, SD)						
BMI (body mass index)	29.49, 8.2	27.59, 7.2	0.245	29.01, 7.9	28.32, 7.64	0.089
Cholesterol (mg/dL)	105.35, 38.7	105.97, 34.7	0.017	105.79, 38.6	104.82, 35.2	0.027
Hemoglobin A1C	5.94, 1.8	5.98, 1.91	0.025	5.88, 1.8	5.96, 1.77	0.043
Visits (N)						
Preventive Medicine Services	18080	43674	0.605	12152	12218	0.004
Emergency Department Services	14321	44830	0.418	10319	10543	0.013
Medications (N)						
Opioid analgesics	31393	69163	1.050	22699	23015	0.017
Benzodiazepine derivative sedatives/hypnotics	26568	43160	1.032	18029	18118	0.005
polyethylene glycol 3350	14902	21806	0.676	8950	9003	0.003
Antipsychotics	8076	4984	0.561	3356	3198	0.015
linaclotide	3791	4033	0.331	1857	1817	0.005
loperamide	3440	3658	0.314	1648	1628	0.003

diphenoxylate	2249	1950	0.262	1036	1052	0.003
lubiprostone	2072	1645	0.256	906	908	< 0.001
Antimuscarinic/antispasmodic combinations	874	555	0.170	389	365	0.006
bismuth subsalicylate	666	722	0.134	328	314	0.004
plecanatide	425	496	0.105	213	211	0.001
eluxadoline	478	346	0.123	204	212	0.003
tegaserod	311	190	0.047	153	139	0.004
prucalopride	355	196	0.053	119	116	0.001
tenapanor	10	10	0.006	10	10	< 0.001

SMD: standardized mean difference; PSM: propensity score matching

Supplementary Data 6. Baseline Demographic and Clinical Characteristics of SNRI users and non-users

	Before PSM			After PSM		
	SNRI users N=60339	Non-users N=332756	SMD	SNRI users N=41256	Non-users N=41256	SMD
Demographics (N)						
Age at Index (mean, SD)	42.55, 11.6	37.06, 13.2	0.442	41.76, 11.7	42.26, 12.06	0.041
Female	49380	220238	0.363	32853	32525	0.020
White	45637	206593	0.296	30626	31026	0.022
Black or African American	4641	22715	0.033	3014	3075	0.006
Hispanic or Latino	3305	19257	0.013	2276	2352	0.008
Asian	588	11186	0.164	495	495	< 0.001
Comorbidities (N)						
Neoplasms	20602	43179	0.515	11599	11860	0.014
Other specified diseases of liver	3500	3983	0.253	1547	1534	0.002
Fibrosis and cirrhosis of liver	958	1145	0.127	380	378	0.001
Hepatic failure, not elsewhere classified	401	330	0.092	148	144	0.002
Chronic hepatitis, not elsewhere classified	257	668	0.040	139	137	0.001
Other specified inflammatory liver diseases	40	36	0.028	11	12	0.001
Type 2 diabetes mellitus	9565	10531	0.443	4440	4454	0.001
Type 1 diabetes mellitus	1678	1564	0.184	653	618	0.007
Chronic ischemic heart disease	3719	3512	0.276	1562	1515	0.006
Heart failure	2220	1550	0.227	822	761	0.011
Atrial fibrillation and flutter	1370	1566	0.155	614	605	0.002
Chronic kidney disease (CKD)	2675	2701	0.228	1118	1116	< 0.001
Nicotine dependence	13034	13813	0.540	6048	5907	0.010
Opioid related disorders	3067	1044	0.298	796	675	0.022
Alcohol related disorders	3492	2577	0.284	1232	1130	0.015
Cannabis related disorders	2742	2186	0.246	954	911	0.007
Personal history of nicotine dependence	7609	7623	0.401	3473	3499	0.002

Generalized anxiety disorder	14004	7057	0.669	4696	4492	0.016
Phobic anxiety disorders	2363	1057	0.252	659	623	0.007
Eating disorders	1701	1036	0.203	537	483	0.012
Specific personality disorders	2362	681	0.263	497	417	0.019
Bipolar disorder	5947	3893	0.388	2002	1870	0.015
Persons with potential health hazards related to socioeconomic and psychosocial circumstances	4391	3160	0.323	1435	1340	0.013
Somatoform disorders	2548	1559	0.250	846	788	0.010
Obsessive-compulsive disorder	1614	749	0.206	468	432	0.008
Major depressive disorder, recurrent, moderate	5873	1231	0.438	1127	941	0.029
Major depressive disorder, recurrent, unspecified	3590	821	0.334	749	599	0.029
Major depressive disorder, recurrent severe without psychotic features	3551	614	0.337	627	480	0.031
Major depressive disorder, recurrent, mild	1915	412	0.241	376	300	0.020
Major depressive disorder, recurrent, severe with psychotic symptoms	666	110	0.143	104	76	0.015
Laboratory values (mean, SD)						
BMI (body mass index)	31.0, 4.5	27.57, 7.2	0.436	30.54, 8.2	28.87, 7.7	0.209
Cholesterol (mg/dL)	106.8, 38.7	105.95, 34.7	0.023	107.91, 38.4	105.85, 35.6	0.056
Hemoglobin A1C	6.02, 1.8	5.98, 1.9	0.023	11307	5.96, 1.7	0.035
Visits (N)						
Preventive Medicine Services	25034	45484	0.655	13166	13238	0.004
Emergency Department Services	20393	45843	0.484	11836	12126	0.015
Medications (N)						
Opioid analgesics	42258	70614	1.124	24982	25752	0.038
Benzodiazepine derivative sedatives/hypnotics	37966	43875	1.193	20695	21161	0.023
polyethylene glycol 3350	18627	22057	0.653	8603	8477	0.008
Antipsychotics	13398	5045	0.675	4132	3836	0.024
linaclotide	5470	4099	0.360	2198	2185	0.001
loperamide	4315	3697	0.307	1590	1549	0.005

diphenoxylate	2752	1966	0.253	1080	997	0.013
lubiprostone	2746	1683	0.260	965	934	0.005
Antimuscarinic/antispasmodic combinations	889	600	0.143	391	375	0.004
bismuth subsalicylate	784	730	0.125	302	266	0.011
plecanatide	605	499	0.113	230	225	0.002
eluxadoline	503	348	0.107	191	186	0.002
tegaserod	332	196	0.089	117	98	0.009
prucalopride	198	190	0.062	87	94	0.004
tenapanor	10	10	0.014	10	0	0.022

SMD: standardized mean difference; PSM: propensity score matching

Supplementary Data 7. Baseline Demographic and Clinical Characteristics of mirtazapine users and non-users

	Before PSM			After PSM		
	Mirtazapine users N=12311	Non-users N=328306	SMD	Mirtazapine users N=8572	Non-users N=8572	SMD
Demographics (N)						
Age at Index (mean, SD)	41.71, 12.48	37.09, 13.16	0.361	40.99, 12.7	41.21, 12.7	0.017
Female	8924	217107	0.138	6042	6006	0.009
White	8717	203355	0.188	5982	6014	0.008
Black or African American	1375	22554	0.151	890	923	0.013
Hispanic or Latino	700	18921	0.003	512	525	0.006
Asian	189	11109	0.120	163	154	0.008
Comorbidities (N)						
Neoplasms	4356	42386	0.544	2568	2622	0.014
Other specified diseases of liver	22	36	0.055	10	10	< 0.001
Fibrosis and cirrhosis of liver	396	1135	0.218	179	183	0.003
Hepatic failure, not elsewhere classified	267	326	0.196	105	105	0.000
Chronic hepatitis, not elsewhere classified	82	667	0.070	43	39	0.007
Other specified inflammatory liver diseases	22	36	0.055	10	10	< 0.001
Type 2 diabetes mellitus	2207	10373	0.495	1122	1191	0.024
Type 1 diabetes mellitus	535	1539	0.255	230	239	0.006
Chronic ischemic heart disease	1098	3428	0.368	502	513	0.005
Heart failure	809	1530	0.336	348	355	0.004
Atrial fibrillation and flutter	490	1538	0.240	225	255	0.021
Chronic kidney disease (CKD)	1038	2670	0.369	469	492	0.012
Nicotine dependence	3822	13539	0.756	1972	2091	0.033
Opioid related disorders	1360	1024	0.477	408	395	0.007
Alcohol related disorders	1482	2513	0.473	573	588	0.007
Cannabis related disorders	1316	2121	0.445	516	508	0.004

Personal history of nicotine dependence	1961	7365	0.490	1006	1040	0.012
Generalized anxiety disorder	3722	7028	0.825	1592	1601	0.003
Phobic anxiety disorders	761	1044	0.335	265	247	0.012
Eating disorders	535	1539	0.255	230	239	0.006
Specific personality disorders	603	1031	0.291	227	226	0.001
Bipolar disorder	2098	3856	0.574	859	828	0.012
Persons with potential health hazards related to socioeconomic and psychosocial circumstances	1482	3090	0.462	537	519	0.009
Somatoform disorders	792	1542	0.331	301	285	0.010
Obsessive-compulsive disorder	509	746	0.270	171	185	0.011
Major depressive disorder, recurrent, moderate	1827	1228	0.567	536	479	0.028
Major depressive disorder, recurrent, unspecified	1286	817	0.465	351	322	0.017
Major depressive disorder, recurrent severe without psychotic features	1477	612	0.510	361	305	0.034
Major depressive disorder, recurrent, mild	554	412	0.294	148	122	0.024
Major depressive disorder, recurrent, severe with psychotic symptoms	306	108	0.221	63	50	0.019
Laboratory values (mean, SD)						
BMI (body mass index)	27.77, 8.3	27.59, 7.2	0.023	27.23, 8.01	28.91, 7.8	0.211
Cholesterol (mg/dL)	99.9, 40.9	105.97, 34.7	0.159	101.41, 40.9	103.2, 36.8	0.046
Hemoglobin A1C	5.96, 1.8	5.98, 1.91	0.014	5.94, 1.8	6.07, 1.8	0.071
Visits (N)						
Preventive Medicine Services	6345	43674	0.895	3609	3766	0.037
Emergency Department Services	3534	44830	0.375	2174	2201	0.007
Medications (N)						
Opioid analgesics	9336	69163	1.310	5869	6068	0.050
Benzodiazepine derivative sedatives/hypnotics	9177	43160	1.575	5605	5750	0.036
polyethylene glycol 3350	5367	21806	0.942	2776	2774	< 0.001
Antipsychotics	4890	4984	1.071	2016	1893	0.034
linaclotide	1717	3658	0.501	694	698	0.002

loperamide	1299	4033	0.404	619	635	0.007
diphenoxylate	886	1950	0.346	390	366	0.014
lubiprostone	712	1645	0.306	315	293	0.014
Antimuscarinic/antispasmodic combinations	241	555	0.175	122	105	0.017
bismuth subsalicylate	282	722	0.187	113	102	0.012
plecanatide	167	190	0.155	77	68	0.011
eluxadoline	145	496	0.127	72	75	0.004
tegaserod	104	346	0.108	57	52	0.007
prucalopride	53	190	0.076	24	25	0.002
tenapanor	10	10	0.038	10	0	0.048

SMD: standardized mean difference; PSM: propensity score matching

Supplementary Data 8. Baseline Demographic and Clinical Characteristics of antispasmodic users and non-users

	Before PSM			After PSM		
	Antispasmodic users N=170797	Non-users N=455177	SMD	Antispasmodic users N=158226	Non-users N=158226	SMD
Demographics (N)						
Age at Index (mean, SD)	37.80, 13.19	37.96, 12.83	0.013	37.60, 13.26	37.36, 13.06	0.019
Female	126015	317166	0.091	115751	114574	0.017
White	123665	300064	0.141	114404	115917	0.021
Black or African American	17722	32994	0.110	15520	15546	0.001
Hispanic or Latino	11913	22560	0.085	10645	10904	0.006
Asian	3299	20329	0.144	3255	2671	0.027
Comorbidities (N)						
Neoplasms	43237	71773	0.238	37163	36947	0.003
Other specified diseases of liver	7200	7160	0.158	5346	4935	0.015
Fibrosis and cirrhosis of liver	1804	2371	0.061	1414	1289	0.009
Hepatic failure, not elsewhere classified	748	842	0.045	586	522	0.007
Chronic hepatitis, not elsewhere classified	528	1004	0.017	442	425	0.002
Other specified inflammatory liver diseases	78	72	0.017	61	52	0.003
Type 2 diabetes mellitus	16316	21898	0.184	13295	12985	0.007
Type 1 diabetes mellitus	2616	3487	0.072	2097	1950	0.008
Chronic ischemic heart disease	6116	7505	0.121	4817	4506	0.012
Heart failure	3437	3667	0.103	2631	2417	0.011
Atrial fibrillation and flutter	2357	2852	0.076	1851	1764	0.005
Chronic kidney disease (CKD)	4628	5570	0.107	3611	3394	0.009
Nicotine dependence	25650	30374	0.271	20879	20248	0.012
Opioid related disorders	4601	3409	0.150	3081	2714	0.017
Alcohol related disorders	6300	7239	0.131	4922	4674	0.009

Cannabis related disorders	5795	4721	0.161	4102	3735	0.015
Personal history of nicotine dependence	14276	16259	0.203	11341	10989	0.009
Generalized anxiety disorder	23128	26336	0.265	18294	17651	0.013
Phobic anxiety disorders	3654	3825	0.107	2805	2624	0.009
Eating disorders	2813	3494	0.081	2223	2166	0.003
Specific personality disorders	2721	3185	0.084	2139	2047	0.005
Bipolar disorder	10673	11449	0.183	8140	7582	0.016
Persons with potential health hazards related to socioeconomic and psychosocial circumstances	7619	7725	0.160	5754	5387	0.013
Somatoform disorders	4247	3937	0.127	3029	2758	0.013
Obsessive-compulsive disorder	2721	3185	0.084	2139	2047	0.005
Major depressive disorder, recurrent, moderate	7617	7726	0.160	5743	5257	0.017
Major depressive disorder, recurrent, unspecified	4527	4909	0.116	3432	3119	0.014
Major depressive disorder, recurrent severe without psychotic features	4226	3992	0.125	3045	2755	0.014
Major depressive disorder, recurrent, mild	2623	2753	0.091	1998	1836	0.009
Major depressive disorder, recurrent, severe with psychotic symptoms	790	734	0.054	568	501	0.007
Laboratory values (mean, SD)						
BMI	29.17, 8.1	28.40, 7.6	28.98,7.9	28.98, 795	28.8, 7.90	0.022
Cholesterol (mg/dL)	106.62, 36.18	106.99, 35.2	106.96, 36.1	106.96, 36.1	106.32, 35.4	0.018
Hemoglobin A1C	5.93, 1.7	6.02, 1.8	5.9, 1.7	5.93, 1.7	5.9, 1.7	0.001
Visits (N)						
Preventive Medicine Services	58188	70920	0.438	48978	48172	0.011
Emergency Department Services	48857	70358	0.321	42443	44350	0.027
Medications (N)						
Opioid analgesics	95029	124833	0.598	82770	82807	< 0.001
Benzodiazepine derivative sedatives/hypnotics	74626	92008	0.520	63174	62554	0.008
polyethylene glycol 3350	40972	38774	0.429	32084	30903	0.019

Antipsychotics	21954	20767	0.297	16606	15456	0.024
linaclotide	8667	8915	0.170	6547	6186	0.012
loperamide	9379	6506	0.224	5993	5332	0.022
diphenoxylate	6014	3568	0.189	3707	3168	0.023
lubiprostone	4352	3780	0.134	3109	2854	0.012
Antimuscarinic/antispasmodic combinations	1919	1325	0.099	1250	1083	0.012
bismuth subsalicylate	2049	918	0.120	1073	871	0.016
plecanatide	1165	903	0.073	778	708	0.006
eluxadoline	1066	530	0.084	549	477	0.008
tegaserod	619	375	0.059	358	296	0.009
prucalopride	524	394	0.050	353	309	0.006
tenapanor	38	10	0.018	19	10	0.006

SMD: standardized mean difference; PSM: propensity score matching

Supplementary Data 9: Active comparison of all-cause mortality risk and hazard ratios among patients with IBS, evaluating antispasmodic users against users of antidepressants, including SSRIs, TCAs, SNRIs, and mirtazapine.

	Before PSM				After PSM			
	Event numbers	Incidence numbers	P-value	Hazard ratio (95% CI)	Event numbers	Incidence numbers	P-value	Hazard ratio (95% CI)
Antidepressant users	189,995	4,271	< 0.001	2.51 (2.25, 2.79)	53,103	773	< 0.001	1.49 (1.32,1.68)
Antispasmodic users	54,947	363		Reference	53,126	376		Reference
SSRI users	125,609	2,655	< 0.001	2.36 (2.11, 2.63)	47,494	685	< 0.001	1.50 (1.32, 1.71)
Antispasmodic users	54,947	363		Reference	47,516	333		Reference
TCA users	45,902	1,180	< 0.001	2.76 (2.45, 3.1)	30,591	536	< 0.001	1.37 (1.18, 1.58)
Antispasmodic users	54,947	363		Reference	30,596	276		Reference
SNRI user	54,425	1,608	< 0.001	3.35 (2.98, 3.76)	28,111	582	< 0.001	1.56 (1.35, 1.81)
Antispasmodic users	50,448	347		Reference	28,109	279		Reference
Mirtazapine users	11,308	682	< 0.001	7.22 (6.35, 8.22)	6,884	332	< 0.001	2.40 (1.93, 2.99)
Antispasmodic users	51,179	351		Reference	6,947	107		Reference

SMD: standardized mean difference; PSM: propensity score matching
